# Interleukin‐4 and Interleukin‐17 are associated with coronary artery disease

**DOI:** 10.1002/clc.24188

**Published:** 2023-12-25

**Authors:** Chenyang Wang, Sheng Liu, Yunxiao Yang, Raimov Kamronbek, Siyao Ni, Yunjiu Cheng, Can Zhou, Huiyuan Yan, Li Li, Hao Liu, Yu Wang, Yanwen Qin, Chengqian Yin, Ming Zhang

**Affiliations:** ^1^ Center for Coronary Heart Disease, Beijing Anzhen Hospital Capital Medical University Beijing China; ^2^ Department of Cardiology, Key Laboratory on Assisted Circulation, Ministry of Health, The First Affiliated Hospital Sun Yat‐Sen University Guangzhou China; ^3^ Department of Cardiology Hangjinqi People's Hospital Inner Mongolia China; ^4^ Liver Research Center, Beijing Friendship Hospital Capital Medical University Beijing China; ^5^ Department of Orthopedics The First Affiliated Hospital of Chongqing Medical University Chongqing China; ^6^ Key Laboratory of Upper Airway Dysfunction‐Related Cardiovascular Diseases, Beijing Anzhen Hospital, Beijing Institute of Heart, Lung and Blood Vessel Disease Capital Medical University Beijing China

**Keywords:** coronary artery disease, cytokines, high‐density lipoprotein cholesterol, interleukin‐4, interleukin‐17

## Abstract

**Introduction:**

The present study aimed to examine the correlation between serum cytokine levels and the incidence of coronary artery disease (CAD), a leading cause of mortality globally, which is known to have a strong association with inflammatory factors. The study further sought to determine the predictors of CAD to distinguish patients with coronary artery lesions from those suspected of having CAD.

**Methods and Results:**

In this study, 487 patients who underwent coronary angiography as a result of suspected CAD but without acute myocardial infarction (AMI) were recruited. The serum levels of the cytokines interleukin (IL)‐1β, IL‐2, IL‐4, IL‐5, IL‐6, IL‐8, IL‐10, IL‐12p70, IL‐17, tumor necrosis factor‐α, interferon (IFN)‐α, and IFN‐γ were measured using a multiplexed particle‐based flow cytometric assay technique. The results of the study revealed that the levels of IL‐4, IL‐12p70, IL‐17, IFN‐α, and IFN‐γ in the CAD group were significantly lower compared to those in the non‐CAD group. Multivariate logistic regression analysis indicated that two serum cytokines (IL‐4 and IL‐17), one protective factor (high‐density lipoprotein cholesterol [HDL‐C]), and three risk factors (sex, smoking, and diabetes) were independently predictive of CAD. The receiver operating characteristic curve analysis showed that the combined use of these predictors in a multivariate model demonstrated good predictive performance for CAD, as evidenced by an area under the curve value of 0.826.

**Conclusion:**

The results of the study indicated that serum IL‐4 and IL‐17 levels serve as independent predictors of CAD. The risk prediction model established in the research, which integrates these serum cytokines (IL‐4 and IL‐17) with relevant clinical risk factors (gender, smoking, and diabetes) and the protective factor HDL‐C, holds the potential to differentiate patients with CAD from those suspected of having CAD but without AMI.

## Graphical abstract

This study included 487 patients who underwent coronary angiography (CAG) as a result of suspected coronary artery disease (CAD) but without acute myocardial infarction (AMI). Cytokine levels were measured. IL‐4 and IL‐17, along with high‐density lipoprotein cholesterol (HDL‐C), sex, smoking, and diabetes, independently predicted CAD. A combined model demonstrated good predictive performance for CAD.

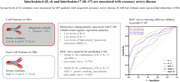

## 1 INTRODUCTION

Coronary artery disease (CAD), a chronic inflammatory condition characterized by the accumulation of fatty deposits in the coronary arteries, is a leading cause of death globally. Despite advances in treatment, which have led to a decrease in CAD mortality rates, the incidence of CAD has been steadily rising.^1^, ^2^ Therefore, identifying predictors of CAD is crucial for early diagnosis and treatment of coronary heart disease. Inflammation, mediated by various proinflammatory cytokines, plays a significant role in the pathogenesis of atherosclerosis. Cytokines that mediate inflammation have also been implicated in various stages of the development of atherosclerosis.^3^, ^4^ Proinflammatory cytokines, such as interleukin‐1β (IL‐1β), interleukin‐6 (IL‐6), tumor necrosis factor‐α (TNF‐α), and interferon‐γ (IFN‐γ), have been implicated in the pathogenesis of CAD.^5^ Elevated levels of IL‐6 have been observed in cases of plaque rupture and may be associated with the susceptibility of atherosclerotic plaques.^6^ The role of interleukin‐4 (IL‐4) and interleukin‐17 (IL‐17) in disease progression and plaque stability is currently a topic of debate, with some studies suggesting a proatherogenic effect, while others propose an atheroprotective role.^7^, ^8^, ^9^ Efforts to develop anti‐inflammatory treatments for atherosclerosis by targeting various proinflammatory cytokines and antiatherosclerosis antibodies have made significant progress in recent years.^10^, ^11^, ^12^

The current study sought to examine the relationship between serum cytokine levels and CAD, with the goal of determining predictors of the disease. The study was designed to exclude patients with acute myocardial infarction (AMI), as AMI has been shown to significantly alter cytokine levels,^13^, ^14^, ^15^ potentially affecting the accuracy of conclusions drawn from previous studies. With a larger sample size compared to many previous studies, the current study aims to establish a clearer understanding of the correlation between cytokines and CAD and to identify CAD patients from those who are suspected of having the disease but have not experienced AMI.

## 2 METHODS

### 2.1 Study population

Between March 12, 2021 and October 10, 2021, individuals suspected of having CAD due to typical or atypical chest discomfort were selected for percutaneous coronary angiography (CAG) at our hospital. All patients selected for the study had not undergone CAG prior. The diagnosis of CAD was established when at least one major coronary artery was found to have severe stenosis (>50%) as determined by CAG analysis. Individuals were excluded from the study if they had a diagnosis of AMI, coronary artery spasm angina, valvular heart disease, active inflammatory or infectious disease, malignant tumor, severe hepatic and renal dysfunction, or autoimmune disease. This study consisted of 487 patients who were divided into two groups based on the results of CAG analysis. The non‐CAD group consisted of 101 individuals with chest discomfort but normal or less severe stenosis (≤50%) in the coronary arteries, while the CAD group consisted of 386 patients with severe stenosis (>50%) in at least one major coronary artery. The CAD group was further subcategorized into stable angina pectoris (SAP) and unstable angina pectoris (UAP) based on clinical symptoms. The study was approved by the Ethics Committee of the Beijing Anzhen Hospital in accordance with the Declaration of Helsinki and authorized by Medical Ethics. Informed consent was obtained from all patients for the use of their serum samples in this research.

Before angiography, a venous blood sample was obtained from each patient. The serum was then separated from the blood sample by centrifugation at 2500*g* for 10 minutes and stored at −80°C for subsequent cytokine analysis.

### 2.2 Demographic and clinical data

The clinical characteristics of all patients, including demographic information such as gender, age, and body mass index, as well as medical histories such as hypertension, hyperlipidemia, diabetes, smoking habits, and use of medications like aspirin, statins, angiotensin‐converting enzyme inhibitors, or angiotensin receptor blockers, were recorded before undergoing CAG. The biochemical indexes were then analyzed in the Biochemical Laboratory of Beijing Anzhen Hospital using a biochemical analyzer (Hitachi‐7600) and a chemiluminescence immunoanalyzer (Abbott‐i2000SR). The analysis was performed with the use of blind quality control specimens, and the intraassay and interassay coefficients of variation were less than 5% and 10%, respectively.

### 2.3 CAG and cytokine assay

All patients underwent CAG after admission and two interventional cardiologists independently diagnosed CAD and calculated the Gensini score (GS) based on the images obtained during the procedure. The serum concentrations of cytokines including IL‐1β, IL‐2, IL‐4, IL‐5, IL‐6, IL‐8, IL‐10, IL‐12p70, IL‐17, TNF‐α, IFN‐α, and IFN‐γ were determined using multichannel particle‐based flow cytometry and analyzed on the BD FACSCanto II system (BD Biosciences) with the aid of a 12‐in‐1 Human Cytokine Assay Kit (Ruisikeer Biotechnology) and FACSCanto clinical Software (BD Biosciences). The analysis was performed following the manufacturer's protocol, and a standard curve was established for each cytokine, with the average fluorescence intensity (mean fluorescence intensity) for each cytokine being converted into concentration using a standard linear portion.

### 2.4 Statistical analysis

The statistical analysis was conducted using SPSS software version 26.0 (IBM SPSS Inc.). The normality of quantitative variables was assessed using the Shapiro–Wilk's test. Quantitative variables with normal distribution were expressed as mean ± standard deviation, while those with non‐normal distribution were expressed as median (25th−75th percentile). Categorical variables were presented as *n* (%). One‐way analysis of variance with post hoc analysis using the Student–Newman–Keuls test or Kruskal–Wallis test with post hoc analysis using Mann–Whitney *U* test was used to compare quantitative variables among the three groups (control, SAP, unstable angina [UA]). To compare two groups of quantitative variables (CAD group and non‐CAD group), Independent Student's *t* tests or Mann–Whitney *U* tests were applied. A comparison of categorical variables was performed using the *χ* ^2^ test. The correlation was determined using Spearman's test due to the nonnormal distribution of variables. Multivariate logistic regression analysis (Model 3: multivariate) was performed to investigate the association between serum cytokines and other risk factors, based on variables with a significance level of *p* < .05 in the univariate test (Model 1: nonadjusted; Model 2: age and sex‐adjusted). Receiver operating characteristic (ROC) curves were used to evaluate the predictive power of IL‐17, IL‐4, and high‐density lipoprotein cholesterol (HDL‐C), and their combination. The performance of each independent variable and the models they combined in predicting CAD was determined by calculating the area under the curve (AUC). All *p* values were two‐tailed, and results were considered statistically significant if *p* < .05.

## 3 RESULTS

### 3.1 Population characteristics

As presented in Table 1, the clinical data comparison between the non‐CAD group and the CAD group showed that the CAD group had a higher proportion of males (*p* < .001), a higher prevalence of smoking (*p* < .001), and diabetes (*p* < .001), as well as higher levels of total triglycerides (TG) (*p* = .001), high‐sensitive C‐reactive protein (hsCRP) (*p* = .002), homocysteine (Hcy) (*p* = .004), serum creatine (Scr) (*p* < .001), fasting blood glucose (FBG) (*p* = .032), and high‐sensitive troponin I (hsTnI) (*p* = .004), and a lower level of HDL‐C (*p* < .001). The comparison of clinical data among the non‐CAD group, SAP group, and UA group revealed that the non‐CAD group had a lower proportion of males (*p* < .01), smoking (*p* < .01), and diabetes (*p* < .01), as well as lower levels of TG (*p* < .01), Scr (*p* < .01), and uric acid (*p* < .05), but a higher level of HDL‐C (*p* < .01) compared to the SAP and UA groups. The levels of hsCRP (*p* < .01), Hcy (*p* < .01), and hsTnI (*p* < .01) were found to be higher in the UA group when compared to the non‐CAD group, but there was no significant difference between the SAP group and the non‐CAD group. The uric acid levels in the SAP group were higher compared to the non‐CAD (*p* < .05) and UA groups (*p* < .05), and the GS increased in increments among these three groups (*p* < .01).

**Table 1:** Clinical characteristics and serum cytokine levels.

	Non‐CAD (*n* = 101)	CA			*p* Value
		SAP (*n* = 53)	UA (*n* = 333)	Total (*n* = 386)	
Clinical characteristics					
Age (years)	59.06 ± 9.56	59.85 ± 10.39	58.33 ± 10.51	58.54 ± 10.49	.617
Male, *n* (%)	44 (43.6)	36 (67.9)^A^	258 (77.5)^A^	294 (76.2)	**<.001**
BMI (kg/m^2^)	25.57 ± 3.39	25.46 ± 2.92	26.11 ± 3.29	26.02 ± 3.25	.226
Smoking, *n* (%)	16 (15.8)	19 (35.8)	142 (42.6)^A^	161 (41.7)	**<.001**
Hypertension, *n* (%)	60 (59.4)	30 (56.6)	209 (62.8)	239 (61.9)	.644
Hyperlipidemia, *n* (%)	80 (79.2)	42 (79.2)	260 (78.1)	302 (78.2)	.833
Diabetes, *n* (%)	18 (17.8)	24 (45.3)^A^	128 (38.4)^A^	152 (39.4)	**<.001**
Aspirin, *n* (%)	46 (45.5)	24 (45.3)	186 (55.9)	210 (54.4)	.112
Statin, *n* (%)	59 (58.40)	31 (58.5)	213 (64.0)	244 (63.2)	.376
TG (mmol/L)	1.27 (0.87–1.75)	1.57 (1.18–2.18)^A^	1.43 (1.10–2.07)^A^	1.44 (1.11–2.08)	**.001**
TC (mmol/L)	3.76 (3.31–4.93)	3.90 (3.03–5.28)	3.70 (3.23–4.30)	3.73 (3.21–4.41)	.124
HDL‐C (mmol/L)	1.13 (0.98–1.28)	0.94 (0.81–1.04)^A^	0.90 (0.79–1.07)^A^	0.91 (0.79–1.07)	**<.001**
LDL‐C (mmol/L)	2.04 (1.68–2.89)	2.11 (1.46–3.48)	1.98 (1.61–2.49)	2.01 (1.60–2.54)	.075
hsCRP (mg/L)	0.83 (0.46–1.65)	1.15 (0.58–2.43)	1.15 (0.67–2.84)^A^	1.15 (0.65–2.76)	**.002**
Hcy (μmol/L)	12.70 (11.30–14.40)	13.45 (12.05–16.05)	13.70 (12.00–15.55)^A^	13.70 (12.00–15.60)	**.004**
Urea (mmol/L)	5.13 (4.09–6.17)	5.51 (4.46–7.03)	5.26 (4.54–6.43)	5.30 (4.51–6.47)	.097
Scr (μmol/L)	64.20 (57.00–73.80)	70.70 (62.15–84.05)^A^	72.60 (64.05–82.85)^A^	72.20 (63.80–82.85)	**<.001**
eGFR (ml/min)	97.74 (90.79–102.64)	93.42 (86.82–102.94)	95.53 (84.51–104.0)	95.26 (84.84–103.90)	.164
Uric acid (μmol/L)	319.10 (266.90–369.30)	368.05 (291.35–425.95)^a^	334.90 (276.75–392.80)^b^	338.3 (277.7–401.1)	.085
FBG (mmol/L)	5.11 (4.75–6.01)	5.32 (4.65–6.59)	5.48 (4.81–6.94)	5.46 (4.78–6.91)	**.032**
hsTnI (pg/mL)	2.60 (1.20–5.20)	3.05 (1.50–7.55)	4.05 (1.80–10.20)^A^	3.80 (1.70–10.1)	**.004**
Gensini score	2 (0–5)	28 (19–40)^A^	46 (22–76)^A^, ^B^	40 (21–70)	**<.001**
Cytokines (pg/mL)					
IL‐1β	1.69 (0.80–3.13)	2.08 (1.03–3.07)	1.65 (0.35–2.90)	1.72 (0.53–2.92)	.539
IL‐2	0.78 (0.44‐1.30)	0.60 (0.45–1.05)	0.67 (0.38–1.08)	0.65 (0.38–1.07)	.410
IL‐4	1.57 (1.06‐2.04)	1.02 (0.62–1.57)^A^	1.09 (0.59–1.55)^A^	1.07 (0.59–1.55)	**<.001**
IL‐5	1.09 (0.51–1.84)	0.97 (0.46–1.47)	0.98 (0.38–1.60)	0.98 (0.42–1.60)	.318
IL‐6	3.81 (2.40–5.99)	3.51 (2.07–6.52)	4.28 (2.58–7.25)	4.06 (2.50–6.98)	.261
IL‐8	37.42 (18.34–73.53)	40.55 (19.67–139.31)	51.85 (23.54–112.15)	49.09 (23.32–112.71)	**.039**
IL‐10	2.05 (1.55–2.91)	2.11 (1.46–2.73)	2.08 (1.43–2.91)	2.09 (1.43–2.86)	.688
IL‐12p70	1.94 (1.15–2.54)	1.94 (1.38–2.58)	1.55 (0.89–2.22)^A^, ^b^	1.57 (0.92–2.29)	**.004**
IL‐17	3.74 (2.49–7.37)	2.29 (0.72–5.72)^a^	2.54 (0.63–5.03)^A^	2.50 (0.66–5.19)	**<.001**
TNF‐α	1.90 (1.05–2.97)	1.44 (0.75–2.52)	1.66 (0.91–2.72)	1.63 (0.86–2.72)	.140
IFN‐α	1.71 (0.99–2.48)	1.48 (0.82–1.98)	1.40 (0.70–2.13)^A^	1.43 (0.71–2.13)	**.003**
IFN‐γ	1.45 (0.82–2.02)	1.13 (0.61–1.64)	1.03 (0.49–1.83)^a^	1.03 (0.49–1.81)	**.011**

*Note*: Values are expressed as percentages, mean ± SD, or median (25th–75th percentile).

Bold values signify the statistical significance in the comparison between the non‐CAD group and the CAD group.

Abbreviations: BMI, body mass index; CAD, coronary artery disease; CI, confidence interval; eGFR, effect glomerular filtration rate; FBG, fasting blood glucose; Hcy, homocysteine; HDL‐C, high‐density lipoprotein cholesterol; hsCRP, high‐sensitive C‐reactive protein; hsTnI, high‐sensitive troponin I; IFN, interferon; IL, interleukin; LDL‐C, low‐density lipoprotein cholesterol; OR, odds ratio; SAP, stable angina pectoris; Scr, serum creatine; TC, total cholesterol; TG, total triglycerides; TNF, tumor necrosis factor; UA, unstable angina.

^A^ *p* < .01 versus non‐CAD group.

^a^ *p* < .05 versus non‐CAD group.

^B^ *p* < .01 versus SAP group.

^b^ *p* < .05 versus SAP group.

### 3.2 Serum cytokine levels of different groups

**Figure 1:**
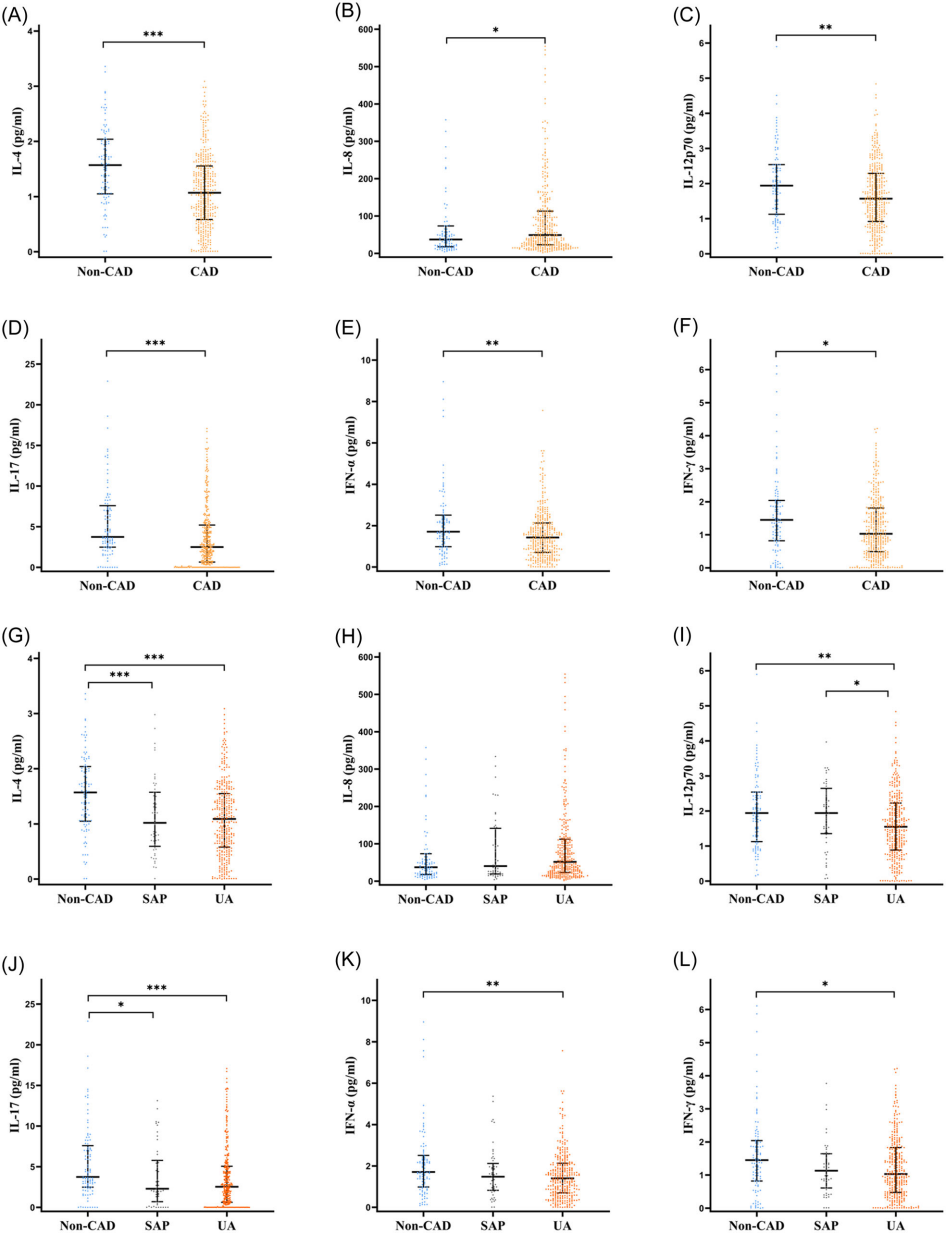
Serum cytokine levels under different groupings. Data are expressed as median with 25th and 75th percentiles. (A–F) Non‐CAD group versus CAD group. (G–L) Non‐CAD group versus SAP group versus UA group. CAD, coronary artery disease; IFN, interferon; IL, interleukin; SAP, stable angina pectoris; UA, unstable angina. *p < .05; **p < .01; ***p < .001.

As indicated in Table 1, there were statistical differences in the levels of six types of serum cytokines, including IL‐4, IL‐8, IL‐12p70, IL‐17, IFN‐α, and IFN‐γ, between the non‐CAD group and the CAD group. These differences in cytokine levels among the different groups were further illustrated in Figure 1. The comparison between the non‐CAD group and the CAD group showed that the CAD group had lower levels of IL‐4 (*p* < .001), IL‐12p70 (*p* = .004), IL‐17 (*p* = .001), IFN‐α (*p* = .003), and IFN‐γ (*p* = .011), while having a higher level of IL‐8 (*p* = .039). Further comparisons among the non‐CAD group, SAP group, and UAP group revealed that the levels of IL‐4 (*p* < .001 and *p* < .001) and IL‐17 (*p* < .001 and *p* < .05) in the non‐CAD group were higher than those in both the UA group and the SAP group. The level of IL‐12p70 in the UA group was lower than both the non‐CAD group (*p* < .01) and the SAP group (*p* < .05), while IFN‐α (*p* < .01) and IFN‐γ (*p* < .05) showed statistical differences between the UA group and the non‐CAD group, but not between the SAP group and the non‐CAD group. The level of IL‐8 had no statistical differences among all three groups.

### 3.3 Correlation between serum cytokine

**Figure 2:**
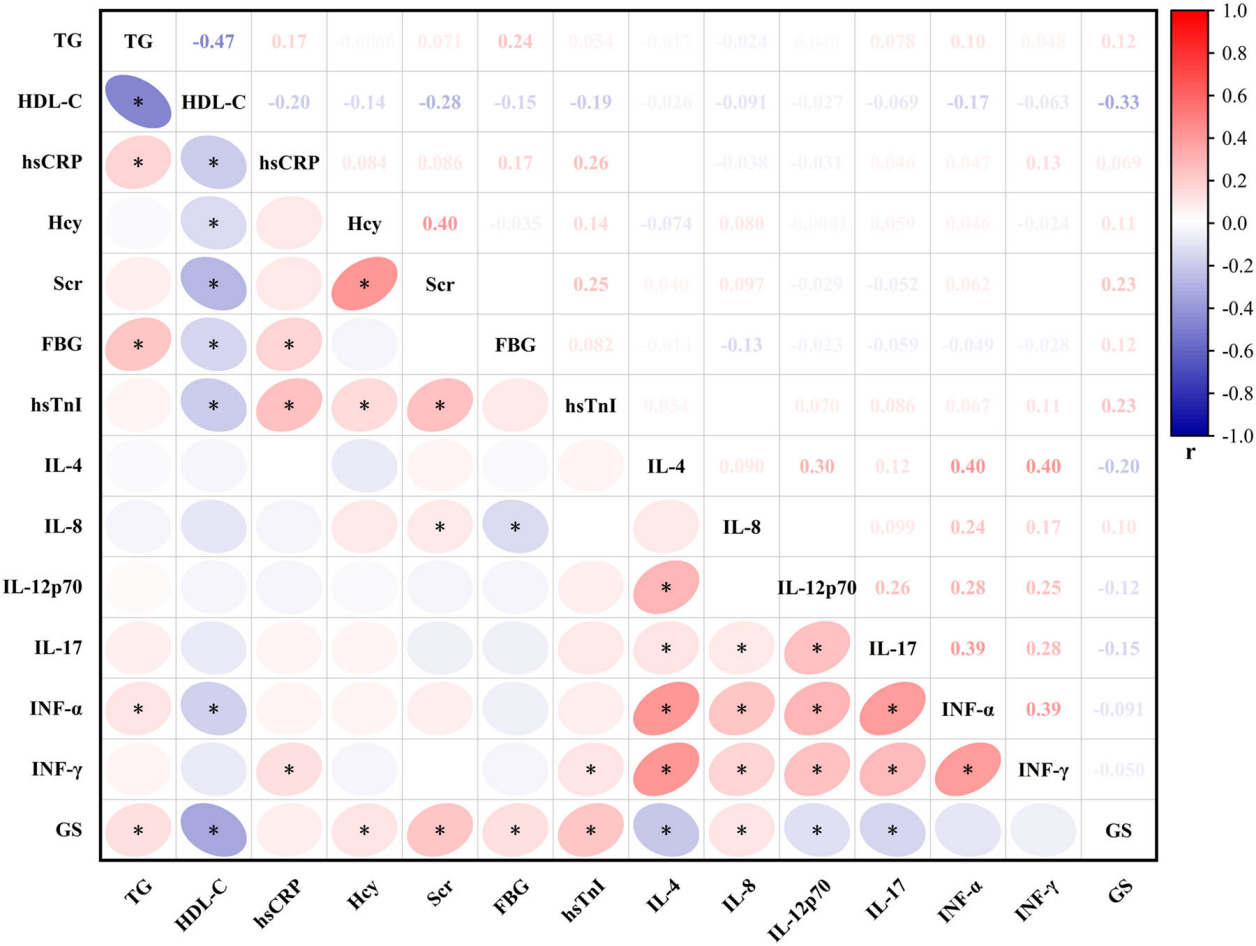
Spearman correlation analysis of variables with statistically significant difference between non‐CAD group and CAD group. The color of the ellipse represents the value of the correlation coefficient. The darker the color of the ellipse, the larger the absolute value of the correlation coefficient; the eccentricity of the ellipse corresponds to the absolute value of the correlation coefficient, with greater eccentricity indicating a stronger correlation; the orientation of the ellipse represents the direction of the correlation. CAD, coronary artery disease; FBG, fasting blood glucose; GS, Gensini score; Hcy, homocysteine; HDL‐C, high‐density lipoprotein cholesterol; hsCRP, high‐sensitive C‐reactive protein; hsTnI, high‐sensitive troponin I; IFN, interferon; IL, interleukin; Scr, serum creatine; TG, total triglycerides. *p < .05.

**Figure 3:**
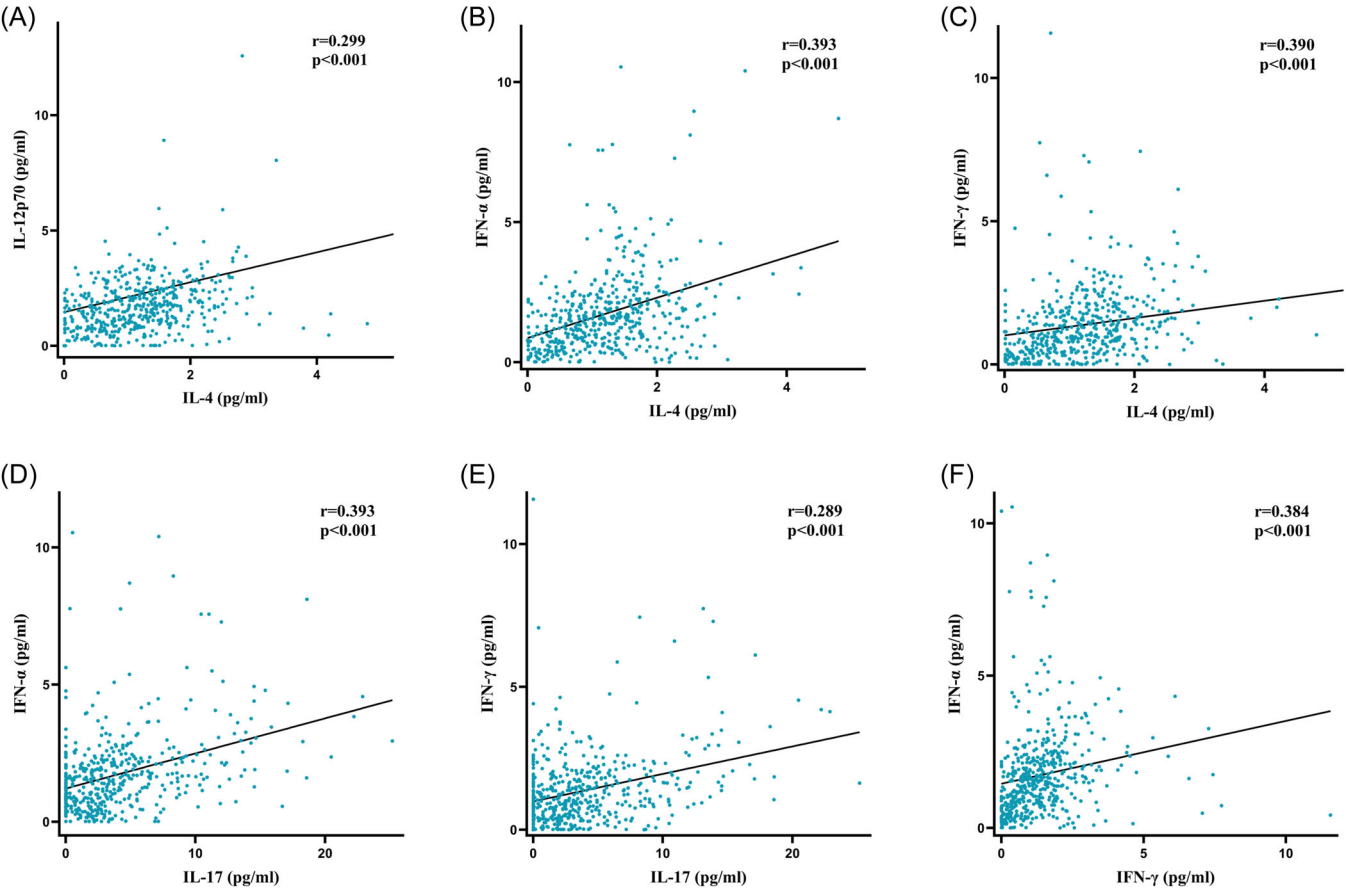
Scatterplots of (A–F) IL‐4, IL‐8, IL‐12p70, IL‐17, IFN‐α, and IFN‐γ. IFN, interferon; IL, interleukin; *r*, correlation coefficient.

The results of a Spearman correlation test, as shown in Figure 2, were utilized to examine the correlations between the serum cytokine levels and other parameters that had previously demonstrated statistical significance between the non‐CAD group and the CAD group in Table 1. The test results indicated the absence of statistically significant correlations between the cytokines and other variables or low correlation coefficients. However, a more considerable positive correlation was observed among these cytokines, as demonstrated in Figure 3. A positive correlation was noted between IL‐4 and the levels of IL‐12p70 (*r* = .299, *p* < .001), IFN‐α (*r* = .393, *p* < .001), and IFN‐γ (*r* = .390, *p* < .001) to varying degrees. Additionally, the levels of IFN‐α (*r* = .393, *p* < .001) and IFN‐γ (*r* = .289, *p* < .001) were positively related to IL‐17, and the level of IFN‐α (*r* = .384, *p* < .001) was positively correlated with IFN‐γ. In addition to the correlation among cytokines, the level of HDL‐C (*r* = −0.32, *p* < .001) was negatively related to GS, and other parameters such as Scr (*r* = .24, *p* < .001), hsTnI (*r* = .23, *p* < .001), and IL‐4 (*r* = −0.17, *p* < .001) displayed a weak correlation with GS.

### 3.4 Multivariate logistic regression analyses of CAD

As shown in Table 2, A logistic regression analysis was conducted to evaluate the impact of various parameters on the prediction of CAD. To maintain the simplicity and accuracy of the model, only variables that were found to be statistically significant in the CAD and non‐CAD groups were included in the further logistic regression analysis. Results from the univariate logistic regression analysis revealed that gender (odds ratio [OR] = 4.140, 95% confidence interval [CI]: 2.619–6.543, *p* < .001), smoking (OR = 3.801, 95% CI: 2.147–6.730, *p* < .001), diabetes (OR = 2.995, 95% CI: 1.730–5.186, *p* < .001), TG (OR = 1.597, 95% CI: 1.189–2.144, *p* = .002), HDL‐C (OR = 0.075, 95% CI: 0.032–0.177, *p* < .001), Scr (OR = 1.035, 95% CI: 1.019–1.053, *p* < .001), FBG (OR = 1.123, 95% CI: 1.005–1.256, *p* = .041), IL‐4 (OR = 0.580, 95% CI: 0.438–0.769, *p* < .001), and IL‐17 (OR = 0.917, 95% CI: 0.875–0.961, *p* < .001) were found to be statistically significant predictors for CAD. After adjusting for age and gender, smoking (OR = 2.180, 95% CI: 1.149–4.134, *p* = .017), diabetes (OR = 3.196, 95% CI: 1.799–5.642, *p* < .001), TG (OR = 1.597, 95% CI: 1.179–2.162, *p* = .002), HDL‐C (OR = 0.116, 95% CI: 0.046–0.289, *p* < .001), FBG (OR = 1.148, 95% CI: 1.024–1.286, *p* = .018), IL‐4 (OR = 0.576, 95% CI: 0.431–0.770, *p* = .007), and IL‐17 (OR = 0.920, 95% CI: 0.875–0.967, *p* = .001) were still found to be correlated with CAD. These variables were then used in the final multivariate logistic regression analysis. Results from the multivariate logistic regression analysis revealed that IL‐4 (OR = 0.534, 95% CI: 0.385–0.741, *p* < .001), IL‐17 (OR = 0.914, 95% CI: 0.863–0.967, *p* = .002), and HDL‐C (OR = 0.148, 95% CI: 0.051–0.433, *p* < .001) were found to be independent predictors for CAD, in addition to gender (OR = 2.296, 95% CI: 1.281–4.115, *p* = .005), smoking (OR = 2.272, 95% CI: 1.151–4.485, *p* = .018), and diabetes (OR = 2.643, 95% CI: 1.313–5.321, *p* = .006) which were identified as classic independent risk factors for CAD.

**Table 2:** Logistic regression analysis to predict CAD.

Variables	Model 1: No adjustment		Model 2: Age and sex‐adjusted		Model 3: Multivariate	
	OR (95% CI)	*p*	OR (95% CI)	*p*	OR (95% CI)	*p*
Age	0.995 (0.973–1.016)	.616	–	–	–	–
Male vs. female	4.140 (2.619–6.543)	**<.001**	–	–	2.296 (1.281–4.115)	**.005**
Smoking	3.801 (2.147–6.730)	**<.001**	2.180 (1.149–4.134)	**.017**	2.272 (1.151–4.485)	**.018**
Diabetes	2.995 (1.730–5.186)	**<.001**	3.196 (1.799–5.642)	**<.001**	2.643 (1.313–5.321)	**.006**
TG	1.597 (1.189–2.144)	**.002**	1.597 (1.179–2.162)	**.002**	1.248 (0.892–1.746)	.195
HDL‐C	0.075 (0.032–0.177)	**<.001**	0.116 (0.046–0.289)	**<.001**	0.148 (0.051–0.433)	**<.001**
hsCRP	1.074 (0.978–1.181)	.136	1.077 (0.975–1.189)	.142	–	–
Hcy	1.029 (0.995–1.065)	.100	1.006 (0.974–1.038)	.722	–	–
Scr	1.035 (1.019–1.053)	**<.001**	1.013 (0.997–1.029)	.122	–	–
FBG	1.123 (1.005–1.256)	**.041**	1.148 (1.024–1.286)	**.018**	1.013 (0.883–1.162)	.857
hsTnI	1.007 (0.997–1.016)	.162	1.005 (0.997–1.013)	.217	–	–
IL‐4	0.580 (0.438–0.769)	**<.001**	0.576 (0.431–0.770)	**<.001**	0.534 (0.385–0.741)	**<.001**
IL‐8	1.000 (1.000–1.001)	.649	1.000 (0.999–1.001)	.787	–	–
IL‐12p70	1.004 (0.974–1.035)	.806	1.001 (0.970–1.034)	.929	–	–
IL‐17	0.917 (0.875–0.961)	**<.001**	0.920 (0.875–0.967)	**.001**	0.914 (0.863–0.967)	**.002**
IFN‐α	0.944 (0.869–1.027)	.181	0.935 (0.858–1.019)	.126	–	–
IFN‐γ	0.869 (0.742–1.018)	.082	0.897 (0.759–1.059)	.200	–	–

*Note*: Bold values signify the statistical significance in the logistic regression analysis to predict CAD.

Model 1: Univariate logistic regression; Model 2: logistic regression after adjusted age and gender; Model 3: parameters with *p* < .05 in Model 2 were subsequently entered into multivariate regression analysis.

Abbreviations: CAD, coronary artery disease; CI, confidence interval; FBG, fasting blood glucose; Hcy, homocysteine; HDL‐C, high‐density lipoprotein cholesterol; hsCRP, high‐sensitive C‐reactive protein; hsTnI, high‐sensitive troponin I; IFN, interferon; IL, interleukin; OR, odds ratio; Scr, serum creatine; TG, total triglycerides.

### 3.5 ROC curve analysis of IL‐4, IL‐17, and HDL‐C in discriminating CAD

**Figure 4:**
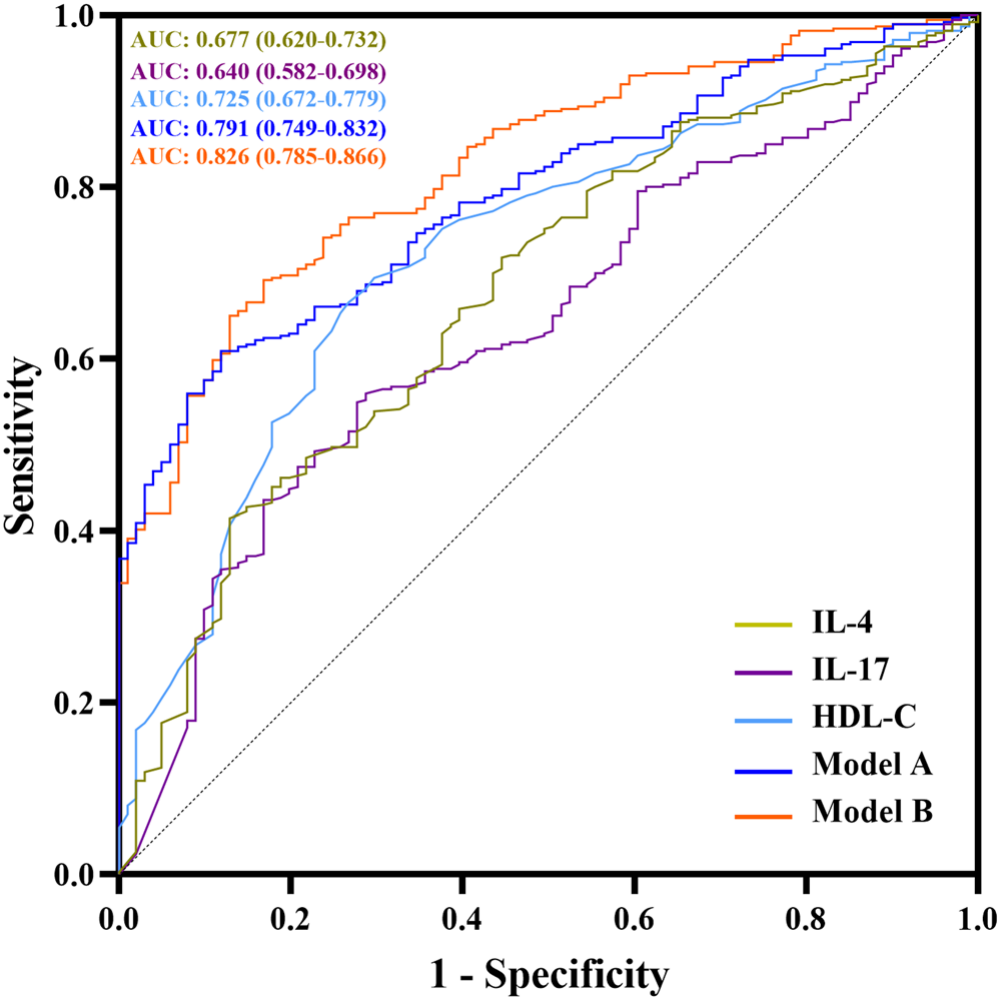
ROC curves of IL‐14, IL‐17, HDL‐C and their combination showing different abilities to predict CAD. Model A: The combination of IL‐4, IL‐17, and HDL‐C; Model B: the combination of IL‐4, IL‐17, HDL‐C, gender, smoking, and diabetes. AUC, area under curve; CAD, coronary artery disease; HDL‐C, high‐density lipoprotein cholesterol; IL, interleukin; ROC, receiver operating characteristic.

The ROC curve was utilized to assess the efficacy of IL‐4, IL‐17, and HDL‐C in differentiating CAD as demonstrated in Figure 4. The AUCs of the ROC curves of IL‐4, IL‐17, and HDL‐C in predicting CAD individually were 0.677 (95% CI: 0.620–0.732, *p* < .001), 0.640 (95% CI: 0.582–0.698, *p* < .001), and 0.725 (95% CI: 0.672–0.779, *p* < .001), respectively. The combination of these three independent factors (Model A) resulted in an increased AUC to 0.791 (95% CI: 0.749–0.832, *p* < .01). Furthermore, the multivariate model (Model B) demonstrated a further improvement in the capacity to predict CAD, with an AUC of 0.826 (95% CI: 0.785–0.866, *p* < .001).

### 3.6 Predictive performance of IL‐4, IL‐17, and HDL‐C for CAD

Further evaluation of the predictive abilities of IL‐4, IL‐17, and HDL‐C in differentiating CAD was carried out by calculating various metrics such as sensitivity, specificity, positive and negative predictive values, false‐positive and false‐negative rates, and positive and negative likelihood ratios, as reported in Table 3. The single‐factor performance of these factors in predicting CAD was observed to be suboptimal, with HDL‐C having the highest sensitivity (66.58%) but low specificity (73.27%) and IL‐4 having the highest specificity (87.13%) but the lowest sensitivity (41.45%). However, the combination of these factors in Model A showed a more comprehensive and improved performance, with a sensitivity of 60.88% and a specificity of 88.12%. This model also exhibited the highest positive predictive value (95.14%) and positive likelihood ratio (5.12), and the lowest negative likelihood ratio (0.44) and false‐positive rate (4.86%). Further integration of gender, smoking, and diabetes in Model B, based on Model A, further enhanced the predictive efficacy of the model, with the highest sensitivity (69.17%) and a relatively high specificity (83.17%). Furthermore, Model B had the highest negative predictive value (41.38%), the lowest false‐negative rate (58.62%), and negative likelihood ratio (0.37).

**Table 3:** Diagnosis accuracy assessment of IL‐4, IL‐17, HDL‐C, and their combination in predicting CAD.

	IL‐4	IL‐17	HDL‐C	Model A	Model B
Sensitivity (%)	41.45 (36.52–46.56)	55.96 (50.84–60.95)	66.58 (61.60–71.23)	60.88 (55.80–65.75)	69.17 (64.26–73.69)
Specificity (%)	87.13 (78.64–92.70)	71.29 (61.30–79.64)	73.27 (63.38–81.36)	88.12 (79.79–93.44)	83.17 (74.13–89.61)
Positive predictive value (%)	92.48 (87.22–95.77)	88.16 (83.28–91.80)	90.49 (86.32–93.53)	95.14 (91.45–97.35)	94.01 (90.41–96.37)
Negative predictive value (%)	28.03 (23.20–33.40)	29.75 (24.15–36.01)	36.45 (29.91–43.52)	37.08 (31.02–43.56)	41.38 (34.60–48.50)
False positive (%)	7.51 (4.23–12.78)	11.84 (8.20–16.71)	9.51 (6.47–13.68)	4.86 (2.65–855)	5.99 (3.63–9.59)
False negative (%)	71.97 (66.60–76.80)	70.25 (63.99–75.84)	63.54 (56.48–70.09)	62.91 (56.44–68.97)	58.62 (51.50–65.41)
Positive likelihood ratios	3.22 (1.91–5.42)	1.95 (1.42–2.68)	2.49 (1.79–3.47)	5.12 (2.99–8.77)	4.11 (2.65–6.37)
Negative likelihood ratios	0.67 (0.62–0.73)	0.62 (0.55–0.70)	0.45 (0.39–0.53)	0.44 (0.39–0.50)	0.37 (0.32–0.43)

*Note*: Data are expressed as *n* (95% confidence interval).

Model A: The combination of IL‐4, IL‐17, and HDL‐C.

Model B: The combination of IL‐4, IL‐17, HDL‐C, gender, smoking, and diabetes.

Abbreviations: CAD, coronary artery disease; HDL‐C, high‐density lipoprotein cholesterol; IL, interleukin.

## 4 DISCUSSION

The prevalence of CAD and the resulting mortality make it imperative to accurately identify individuals with the disease. The present study aimed to investigate the correlation between serum cytokine levels and CAD and to establish a novel prediction model for the diagnosis of CAD. Results indicated a significant difference in serum cytokine levels between CAD patients and non‐CAD patients, with lower levels of IL‐4 and IL‐17 being associated with CAD after adjusting for other relevant factors. The prediction model, which was developed by integrating serum IL‐4, IL‐17, HDL‐C, and other cardiovascular risk factors, demonstrated a high degree of accuracy in differentiating patients with CAD from suspected CAD patients, with a sensitivity and specificity of 69.17% and 83.17%, respectively, as indicated by ROC curve analysis.

In our study, we excluded patients with AMI for two reasons. First, the development of atherosclerosis, which was the underlying pathological process for CAD, was a gradual process. AMI, on the other hand, was a special and acute form of CAD and resulted in significant changes in serum cytokine levels, reflecting the acute plaque rupture and myocardial injury, and it differed from slow changes in cytokines during the process of atherosclerosis.^15^, ^16^, ^17^ The inclusion of AMI patients in the study cohort to predict CAD could result in inaccurate findings. Second, the results of studies involving AMI patients were not applicable to clinical practice. Even in primary healthcare institutions, AMI could be accurately diagnosed using electrocardiogram, troponin, and typical symptoms of chest pain. Troponin had a high sensitivity and specificity for the diagnosis of myocardial infarction, which surpasses the diagnostic ability of cytokines.

In our study, CAG was utilized as the gold standard for diagnosing CAD and the suspected CAD population was divided into CAD and non‐CAD groups based on the results. The levels of IL‐4, IL‐12p70, IL‐17, IFN‐α, and IFN‐γ were found to be significantly lower in the CAD group compared to the non‐CAD group, while the level of IL‐8 was significantly higher. The role of IL‐17 in the development of atherosclerosis was a matter of debate, as some studies had indicated a proatherosclerotic effect, while others had reported antiatherosclerotic properties.^18^, ^19^, ^20^ Ablation of IL‐17 or its receptor had been found to reduce lesion development and plaque vulnerability in atherogenic mouse models,^21^, ^22^, ^23^ which supported a proatherosclerotic effect of IL‐17. However, there was also evidence suggesting that IL‐17 might contribute to the induction of a stable plaque phenotype, which was linked to plaque stability.^20^, ^24^ The same was true for IL‐4, where some studies had indicated a protective effect against atherosclerosis,^25^, ^26^ while others had not found any support for an atheroprotective role for IL‐4.^27^, ^28^ In addition, IL‐4 had been shown to promote the expression of other inflammatory mediators, which might contribute to the development of atherosclerosis.^29^, ^30^, ^31^ The lower levels of IL‐4 and IL‐17 in the CAD group, as compared to the non‐CAD group in our study, suggested that these cytokines might have a protective effect against atherosclerosis and that high levels of IL‐4 and IL‐17 might indicate false‐positive coronary lesions in patients suspected of having CAD.

The results of this study align with previous research indicating a strong correlation between high levels of IL‐8 and atherosclerosis.^32^, ^33^ In regard to IL‐12p70, previous studies have shown it to be a proinflammatory cytokine contributing to the development and enlargement of atherosclerotic plaque, and to have higher concentrations in individuals with CAD.^28^, ^34^, ^35^, ^36^ However, the present study found lower levels of IL‐12p70 and IFN‐γ in the CAD group compared to the non‐CAD group. This discrepancy could be attributed to the exclusion of patients with AMI, as previous studies have reported significantly higher levels of IL‐12p70 and IFN‐γ in AMI patients compared to those without AMI.^16^, ^37^, ^38^ IFN‐α, a pluripotent inflammatory cytokine, was believed to play a role as an amplifier of inflammation within the atherosclerotic plaque.^39^ However, there have been few studies that have examined changes in the levels of IFN‐α in patients with CAD. The present study found that the level of IFN‐α was lower in the CAD group compared to the non‐CAD group. The results of Spearman correlation analyses showed a significant relationship between the levels of IL‐12p70, IFN‐α, IFN‐γ, IL‐4, and IL‐17, suggesting that these cytokines may have synergistic effects in the development of CAD.

To determine the accuracy of serum cytokines in predicting CAD, univariate and multivariate logistic regression analyses were conducted. The results of the analysis revealed that two serum cytokines (IL‐4 and IL‐17), three clinical risk factors (gender, smoking, and diabetes), and HDL‐C were independently associated with CAD after controlling for age, gender, and other related factors. While the ideal predictors for clinical use should have high sensitivity and specificity, the use of a single biomarker to identify complex diseases typically results in poor sensitivity.^40^ In this study, when IL‐4, IL‐17, and HDL‐C were used alone to predict CAD, the results had high specificity but low sensitivity. To enhance the prediction performance, two models (A and B) were developed by combining IL‐4, IL‐17, and HDL‐C. The sensitivity was improved to 60.88% and 69.17%, respectively, in Model A and Model B, while retaining a good specificity of 83.17% in Model B.

While angiography remains the gold standard for diagnosing CAD, our proposed predictive model, in combination with routine clinical tests, could offer a cost‐effective and low‐risk option for guiding the diagnosis of suspected CAD patients, especially in primary care facilities where access to angiography resources may be limited. Our model has the potential to spare non‐CAD patients from undergoing unnecessary angiography, reducing the financial burden and exposure to ionizing radiation. Meanwhile, patients with CAD identified by the model could be promptly referred to specialized hospitals for further evaluation and treatment, including CAG.

It is important to acknowledge the limitations of our study. First, while our study focused on 12 cytokines, there may be other cytokines that play a role in atherosclerosis and CAD. Second, our study was a cross‐sectional study without follow‐up data, and further prospective studies are needed to confirm the potential of serum cytokines in diagnosing CAD. Third, previous studies have shown the association between IL‐1β, IL‐6, and TNF‐α and CAD, however, our study did not find a similar association which may be due to the exclusion of AMI patients and the limited sample size. Furthermore, while the sample size in this study was relatively large, it still falls short in comparison to the overall CAD population. Finally, the study was conducted at a single center and primarily involved patients from northern China, further multicenter research in different regions would enhance the credibility of the findings.

In conclusion, our study found that serum IL‐4 and IL‐17 levels were significantly associated with the presence of CAD. The risk prediction model constructed by combining these serum cytokines with other clinical risk factors such as gender, smoking, diabetes, and HDL‐C, has the potential to identify individuals with CAD among those who have suspected CAD but not AMI. This model could serve as a cost‐effective and low‐risk diagnostic tool to guide the referral of symptomatic patients for angiography, especially in resource‐limited settings.

## AUTHOR CONTRIBUTIONS

Chenyang Wang, Sheng Liu, Chengqian Yin, and Ming Zhang conceived and designed the study. Chenyang Wang, Siyao Ni, Yunxiao Yang, and Can Zhou collected clinical data from patients. Sheng Liu, Yunjiu Cheng, Huiyuan Yan, and Li Li analyzed the data. Yanwen Qin, Chengqian Yin, and Ming Zhang provided significant input to the manuscript. Sheng Liu, Chenyang Wang, Hao Liu, and Yu Wang wrote the manuscript with input from all authors. All authors contributed to the article and agree to be accountable for the content of the work.

## Data Availability

The datasets presented in this article are not readily available because of the protection of personal data and privacy restrictions. Requests to access the datasets should be directed to Ming Zhang, zhangming2279@hotmail.com.
